# Implicit video feedback produces positive changes in landing mechanics

**DOI:** 10.1186/s40634-018-0129-5

**Published:** 2018-05-02

**Authors:** Tijana Popovic, Shane V. Caswell, Anne Benjaminse, Tarique Siragy, Jatin Ambegaonkar, Nelson Cortes

**Affiliations:** 1 Sports Medicine Assessment, Research & Testing (SMART) Laboratory, George Mason University, 10890 George Mason Circle Bull Run Hall 220, MSN 4E5, Manassas, VA 20110 USA; 2 Department of Radiology and Biomedical Imaging, University of California, 185 Berry Street, Lobby 6, Suite 391, San Francisco, CA 94107 USA; 3 University of Groningen, 9712 Groningen, CP Netherlands; 4 School of Human Kinetics, Faculty of Health Sciences at the University of Ottawa, 75 Laurier Avenue East, Ottawa, ON K1N 6N5 Canada

**Keywords:** Implicit and explicit feedback, Drop-jump, ACL injury prevention, Lower extremity mechanics

## Abstract

**Background:**

Implicit (IF) and explicit (EF) feedback are two motor learning strategies demonstrated to alter movement patterns. There is conflicting evidence on which strategy produces better outcomes. The purpose of this study was to examine the effects of reduced IF and EF video feedback on lower extremity landing mechanics.

**Methods:**

Thirty participants (24 ± 2 years, 1.7 ± 0.1 m, 70 ± 11 kg) were randomly assigned to three groups: IF (*n* = 10), EF (n = 10), and control (CG) (n = 10). They performed twelve box-drop jumps three times a week on the training sessions for six weeks. Only IF and EF groups received video feedback on the training sessions. IF was cued to focus their attention on the overall jump, while EF was cued to focus on position of their knees. 3D lower extremity biomechanics were tested on testing sessions with no feedback. All sessions were at least 24 h apart from another. Testing sessions included baseline testing (pretest), testing after 3 training sessions with 100% feedback (pst1), testing after 6 training sessions with 33.3% feedback (pst2), testing after 6 training sessions with 16.6% feedback (Pst3), and testing 1 month after with no feedback (retention – ret). ANOVA compared differences between groups and time at initial contact and peak for hip flexion (HF, **°**) and abduction angle (HA, **°**), hip abduction moment (HAM, Nm/kgm), knee flexion (KF, **°**) and abduction angle (KA, **°**), knee abduction moment (KAM, Nm/kgm) and VGRF (N) (*p* < 0.05).

**Results:**

A significant main effect for group was found between IF and EF groups for HA (IF = − 6.7 ± 4; EF = − 9.4 ± 4.1) and KAM (IF = 0.05 ± 0.2; EF = − 0.07 ± 0.2) at initial contact, and peaks HA (IF = − 3.5 ± 4.5; EF = − 7.9 ± 4.7) and HAM (IF = 1.1 ± 0.6; EF = 0.9 ± 0.4). A significant main effect for time at initial contact for HF (pre = 32.4 ± 3.2; pst2 = 36.9 ± 3.2; pst3 = 37.9 ± 3.7; ret. = 34.1 ± 3.7), HAM (pre = 0.1 ± 0.1; pst1 = 0.04 ± 0.1; pst3 = 0.1 ± 0.01), KA (pre = 0.7 ± 1.1; pst1 = 0.2 ± 1.2; pst3 = 1.7 ± 1), and KAM (pre = 0.003 ± 0.1; pst3 = 0.01 ± 0.1) was found.

**Discussion/conclusion:**

We found that implicit feedback produced positive changes in landing mechanics while explicit feedback degraded motor learning. Our results indicate that implicit feedback should be used in programs to lower the ACL injury risk. We suggest that implicit feedback should be frequent in the beginning and not be reduced as much following the acquisition phase.

## Background

Approximately 200,000 anterior cruciate ligament (ACL) injuries occur in the US annually (Paterno et al. 2014) resulting in more than 2.5 billion dollars spent on ACL reconstruction surgery. ACL injury can have short term repercussions such as delayed return to sport participation and physical activity reduction (Murray et al. 2013). As well as detrimental long-term outcomes (e.g., development of knee osteoarthritis), (Filbay et al. 2015) and reduced quality of life (Gottlob et al. 1999). More than 70% of all ACL injuries are noncontact (Kim et al. 2015). The most common mechanisms of noncontact ACL injury include sudden change of direction, (Jamison et al. 2013) rapid deceleration and acceleration, (Laskowski 2014) and stiff-legged landing after a jump, (Myklebust and Steffen 2015) leading to increased hip abduction angle, Chaudhari and Andriacchi 2006) knee abduction moment, (Myklebust and Steffen 2015) and decreased knee flexion angle (Myklebust and Steffen 2015). Despite several ACL injury prevention programs to alter risk factors, (Hübscher et al. 2010) the rate of noncontact ACL injury has remained steady for the past decade (LaBella et al. 2014).

Neuromuscular training has been shown to reduce ACL injury risk factors during landing (Kruse et al. 2012; Myklebust and Steffen 2015). These programs have focused on increasing muscle strength, dynamic joint stability, and improving individuals’ awareness of proper movement patterns during various activities.(Risberg et al. 2001; Kamper and Moseley 2011). Feedback may be added to augment skill learning or modification. Augmented feedback via external sources (e.g., video, instructor, teacher)(Benjaminse et al. 2010) is an important component of neuromuscular ACL injury prevention training programs to decrease injury risk (Chaudhari and Andriacchi 2006). ACL injury risk factors changed the most when video feedback is used in conjunction with verbal feedback (Hewett 2005; Benjaminse et al. 2015). For instance, video and verbal feedback has been reported to decrease vertical ground reaction force (vGRF) and increase peak knee flexion angle during a drop jump task (Hewett 2005; Gokeler et al. 2015).

Implicit and explicit instructions are two modes of augmented instructions that facilitate motor learning (Benjaminse et al. 2010; Pascua et al. 2014; Gokeler et al. 2015). Implicit refers to the automatic acquisition of a motor skill whereby a participant’s attention is directed to an external focus (outcome or effect) (Benjaminse et al. 2010). An example of implicit instruction would be to mention *“imagine kicking a ball”, “to facilitate extension of the knee.*”^11^ Explicit instruction is the process whereby participant’s attention is consciously directed toward an internal focus (participant’s own movement patterns); for example, stating to “*keep the knees over the toes”*^11^ Previous research suggests that implicit and explicit feedback could influence both immediate outcome and short-term changes in motor learning (Munzert et al. 2014; Pascua et al. 2014). Most neuromuscular ACL injury prevention programs contain explicit instructions for performing correct movement patterns while landing (e.g., position of trunk, hips, knees, and feet) (Noyes et al. 2005). However, directing attention to one’s own mechanics may disrupt automatic motor processes resulting in a detriment on motor learning and performance (Wulf et al. 2002; Wulf 2013).

In addition, skills acquired through implicit feedback are retained longer, are more resistant to stress, and do not degrade in the presence of physiological fatigue compared to explicitly acquired skills (Wulf and Prinz 2001; Benjaminse et al. 2010). For example, it has been reported that explicit instruction produces immediate positive change, that is not maintained in the retention phase (Maxwell et al. 2000; Benjaminse and Otten 2011). McNair et al. reported that 80 healthy individuals improved their landing technique using implicit and explicit feedback, with implicit group reporting better results than explicit and control in the retention (McNair et al. 2000). Prior research reported that individuals with ACL reconstruction had greater increase in peak knee flexion, time to peak knee angles, and ROM in both legs when provided implicit feedback than those provided with explicit feedback (Gokeler et al. 2015).

Facilitating motor learning and retention through feedback modes depends on the frequency of feedback provided to participants (Wulf et al. 2010; Pascua et al. 2014). Guidance hypothesis states that too frequent feedback negatively affects learning (Park et al. 2000; Anderson et al. 2005; Schmidt 1991). However, by gradually decreasing the feedback frequency, acquired motor skills do not diminish as quickly in the absence of feedback (Park et al. 2000; Anderson et al. 2005).

Both implicit and explicit feedback strategies have been utilized for injury prevention. Substantial amount of research indicates that implicit feedback is superior in facilitating motor learning in the retention. However, prior research primarily focused on finding the optimal explicit feedback frequency with very limited research on implicit feedback. The optimal amount and frequency of implicit feedback linked to greatest improvement in transfer and retention is still unknown. Therefore, the purpose of this study is to test the effects of reduced implicit and explicit video feedback on lower extremity landing mechanics during different phases in motor learning and to determine if those changes are constant over time even when feedback is not provided.

We hypothesized that the implicit group will increase hip flexion and abduction and knee flexion angle and decrease hip and knee abduction moment and vGRF when compared to the explicit group. Additionally, we hypothesized that both implicit and explicit groups will reduce landing patterns reported to be risk factors for ACL injury when compared to the control group.

## Methods

### Participants

**Fig. 1:**
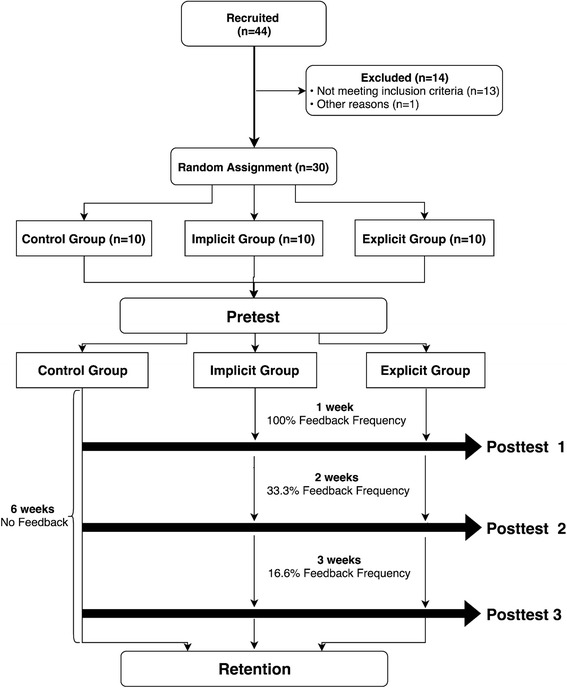
Consort flow chart of study design

A randomized controlled trial design was used in this study and is illustrated in Fig. 1. Dependent variables included kinematic (hip and knee flexion and abduction angle) and kinetic measurements (hip and knee abduction moment and vGRF) at both peak and initial contact during the first landing. A priori sample size was estimated from previous research using an α value of 0.05 and power of 80% (Etnoyer et al. 2013). Thirty healthy individuals 18–35 years old (IF: 25 ± 3 years; 1.72 ± 0.1 m; 69 ± 12 kg; EF: 23 ± 2 years; 1.74 ± 0.1 m; 74 ± 8 kg; CG: 24 ± 2 years; 1.78 ± 0.1 m; 68 ± 14 kg) volunteered to participate in this study (Table 1). The testing was performed at George Mason University in Manassas, VA. The university’s Human Subjects Review Board approved the study (642920–1). All participants signed informed consent prior to participation. The inclusion criteria: i) 18–35 years old, ii) exercise at least 3-days/week for a minimum of 20-min (Oñate et al. 2005). Participants were excluded if they had: i) a lower extremity injury 6-months prior to testing, ii) knee surgery, iii) self-reported lower extremity instability at the time of the study, iv) other known lower extremity impairments, and/or v) a history of participation in supervised lower extremity injury prevention programs. Injury was defined as any musculoskeletal complaint that stopped the participant from undertaking their normal exercise routine (Munro 2013).

**Table 1:** Participants’ demographics (age, height, mass: mean ± standard deviation)

	Age (yrs)	Height (m)	Mass (kg)
Implicit group (*n* = 10)	25 ± 3	1.72 ± 0.1	69 ± 12
Explicit group (*n* = 10)	23 ± 2	1.74 ± 0.1	74 ± 8
Control group (*n* = 10)	24 ± 2	1.78 ± 0.1	68 ± 14

Participants were instructed to wear spandex or lycra compression shorts, no shirt (men) and sports bra (women) and tennis shoes they used for normal exercise. Primary investigator randomly assigned participants to one of three groups (implicit group-IF, explicit group-EF, and control group-CG) by asking participants to select one of three colored envelopes (green = IF, red = EF, blue = CG). Participants were blinded for dependent outcomes or digital graphs investigator used to determine the best jump in IF.

### Procedures

An 8-camera VICON motion capture system sampling at 200 Hz was used to collect trajectory data (Vicon Motion Analysis Systems Inc., Oxford, UK). VICON system validity and reliability has been previously reported (McGinley et al. 2009). Two force plates (Bertec Corporation, Worthington, OH) set at 1000 Hz were used to collect ground reaction force data. Data from force plates were used to determine the initial contact point (GRF > 10 N).

A stadiometer and digital scale were used to measure height (meters) and body mass (kilogram). Forty-four reflective markers (34 tracking and 10 calibration) were placed on each participant’s lower extremity landmarks using double sided tape (Cortes et al. 2014). A single tracking marker was placed on each posterior-superior iliac crest and anterior iliac crest. A 5-marker cluster was placed on each thigh and foot. Finally, a 4-marker cluster was placed on each shank. Athletic and powerflex tape were used to secure marker positions. The 10 calibration markers were placed on the greater trochanters, medial and lateral femoral condyles, and medial and lateral malleoli. Participants were allowed 5-min self-selected warm up. They were then instructed to stand on a force plate in the anatomical position with feet shoulder width apart. A static standing trial and a functional hip motion trial were then obtained. Functional hip calibration was used to estimate hip joint center (Cortes et al. 2012). The calibration markers were then removed. After calibration markers were removed, the task was explained to the participant.

General verbal instructions were provided to each group on how to perform the box-drop jump. The box was 30 cm in height and placed 30 cm away from the force plates (Cortes et al. 2007; Etnoyer et al. 2013). The general instructions were: “*A 30cm tall box is placed exactly 30cm from the force plates. Drop from the box onto the two closest force plates. Upon contacting the force plates immediately perform a maximal vertical jump. At no point, should your feet touch the two further force plates. Before jumping, be sure that you are mentally prepared to proceed*.” All participants were allowed 3 practice trials to familiarize themselves with the task; no data were collected on these trials. Following the practice trials, participants engaged in 5 pretest trials to collect baseline values. Participants were allowed up to 2 min of rest in between trials. No further instruction or feedback was provided for the practice or pretest trials. Trials were discarded if both feet did not land on the corresponding force plate or if participants lost balance. After baseline values were collected (pretest), participants in the experimental groups received the intervention portion of the study on separate days over 6 weeks.

All participants were instructed to come to the laboratory 3 times/week for a total of 20 sessions. The first (pre), fifth (pst1), twelfth (pst2), ninetieth (pst3), and twentieth (ret) sessions were testing sessions. Each testing session contained 5 jumps and no feedback was provided to any group. Between testing sessions all groups had intervention sessions. The intervention sessions were 2nd-4th, 6th–11th, 13th–19th (Fig. 1). During the intervention sessions, all participants performed 12 jumps but video feedback was provided only to the experimental groups (IF and EF). Two Sony HDR-CX380 camcorders were used to provide feedback to experimental groups. The first camcorder was placed on the left-hand side of the participant and captured the sagittal plane view from a height of 94 cm. The second camcorder faced the participants and captured the frontal plane view from a height of 107 cm. Both camcorders were placed 2 m from the participants and were mounted on tripods. Participants in the experimental groups then received video feedback that consisted of sagittal and frontal plane recordings of themselves performing the task. This allowed participants to analyze their own jumping mechanics. Participants viewed 2 video recordings per camera; once in real-time and once in slow motion (5× slower) during the 2-min rest period. Before participants viewed the videos, the investigator provided a verbal cue to participants.

Participants in the implicit group were instructed to focus on their result: “*There is a superior and inferior way of performing the task. The task you see now is the best performance so far. While watching the video self-assess how to achieve the best performance, concentrate on your whole-body movement, especially during landing. During the next jump, try to mimic the jump as best as possible.*” The principal investigator determined the best performance based on each participant’s knee abduction angle which was set at a threshold between neutral frontal plane knee alignment (0°) and − 5°.

The explicit group was provided video feedback but with distinct verbal instructions to focus on “*When watching the video of your performance, focus if you have knocked knees, bowed leg stance, and shallow knee flexion angle. During the next jump, try to focus on not having knocked knees or bowed leg stance, and have deeper knee flexion angle.*” In the first week after the pretest (pre), participants in experimental groups received 100% of feedback (after every jump). Feedback frequency was then lowered to 33.3% (every third jump) for the next 2 weeks. For the last 3 weeks, feedback frequency was reduced to 16.6% (every sixth jump).

### Data processing and statistical analyses

Data collected from the standing trial was used to create a kinematic model of the lower extremity (pelvis, thigh, shank, and foot) using Visual 3D (C-Motion, Germantown, MD, USA) with a least-squares optimization (Cortes et al. 2012). This kinematic model was used to quantify hip, knee and ankle joints motion. Using segment inertial characteristics, a standard inverse dynamic was created to calculate 3-D joint forces and moments from the kinematic and ground force data (Cortes et al. 2012). All trajectory and force plate data were passed through a fourth-order, zero-phase lag, low-pass Butterworth filter with a cutoff frequency of 7 Hz and 25 Hz, respectively (Cortes et al. 2014). Visual 3D software was used to calculate three-dimensional joint rotations and moments. Joint rotations were quantified based on the position of the distal segment relative to the proximal segment. All joint moments were normalized to each participant’s mass and height (Nm/kg). Data for dependent variables were exported from Visual 3D into Excel (Microsoft Corporation, Redmond, WA). Peak values for all testing trials were averaged for each time point and exported into SPSS (IBM Corporation, Armonk, NY) for data analysis. We conducted a generalized linear model ANOVA 3 (group) × 5 (time point during testing sessions) with Bonferroni correction. An alpha level was set a priori at 0.05.

## Results

No statistically significant differences were found between groups for age, mass, and height at pretest. Further, no significant interaction between group and time (*p* > 0.05) for any dependent measure was observed.

A statistically significant main effect for time across all groups was observed for the following variables at initial contact: hip flexion angle, hip abduction moment, knee abduction, and knee abduction moment (*p* ≤ 0.05; Tables 2 & 3). Hip flexion increased from pretest to posttest-2 and to posttest-3 (*p* = 0.02). In addition, hip flexion decreased from posttest-3 to retention test (*p* = 0.02). Furthermore, hip abduction moment decreased from pretest to posttest-1 and then increased from posttest-1 to posttest-3 (*p* = 0.01). Likewise, knee abduction angle increased from pretest to posttest-3 (*p* = 0.03) and from posttest-1 to posttest-3 (*p* = 0.01). Lastly, knee abduction moment increased from posttest-1 to posttest-3 (*p* = 0.04).

**Table 2:** Descriptive statistics for all dependent measures by group across testing periods measured at initial contact

Box Drop-Jump Task	Pretest		Posttest 1		Posttest 2		Posttest 3		Retention	
	Mean ± SD	95% CI	Mean ± SD	95% CI	Mean ± SD	95% CI	Mean ± SD	95% CI	Mean ± SD	95% CI
HF (°)										
IG	32.6 ± 12.4	26, 39.2	35 ± 10.4	28.7, 41.2	36.9 ± 11.2	30.5, 43.4	38.5 ± 13.9	30.9, 46	31.8 ± 16.3	24.2, 39.5
EG	31.8 ± 9.6	25.2, 38.4	34.1 ± 10.1	27.9, 40.4	35.9 ± 9	29.4, 42.3	37.2 ± 11.4	29.6, 44.8	34.9 ± 6.8	27.3, 42.5
CG	34.1 ± 7.9	27.5, 40.7	36.9 ± 8.1	30.6, 43.1	37.9 ± 9.6	31.4, 44.4	38 ± 9.3	30.5, 45.6	35.6 ± 10.1	27.9, 43.2
HA (°)										
IG	−6.78 ± 3.6	−9.8, −3.8	−5.7 ± 2.6	−8.2, − 3.3	− 6.7 ± 5	− 9.9, − 3.6	−7.9 ± 5.3	−11.4, − 4.4	− 6.5 ± 3.9	−9.7, − 3.3
EG	− 9.2 ± 4.8	− 12.2, − 6.2	− 8.4 ± 4.5	−10.8, − 6	−9.8 ± 2.8	− 12.9, − 6.6	−9.2 ± 4	−12.8, − 5.7	− 10.6 ± 4.5	− 13.8, − 7.4
CG	− 8.7 ± 5.3	− 11.7, − 5.7	− 8.4 ± 3.7	−10.8, − 6	−7.3 ± 6.2	−10.5, − 4.2	−7.8 ± 6.7	−11.3, − 4.3	−7.5 ± 6.2	−10.7, − 4.3
HAM (Nm/kg)										
IG	0.003 ± 0.4	−0.3, 0.3	0.1 ± 0.4	− 0.2, 0.3	0.01 ± 0.4	− 0.3, 0.3	0.1 ± 0.4	− 0.1, 0.3	0.01 ± 0.4	− 0.2, 0.2
EG	0.03 ± 0.4	−0.2, 0.3	−0.1 ± 0.3	− 0.3, 0.1	−0.1 ± 0.5	− 0.4, 0.2	− 0.1 ± 0.4	− 0.3, 0.1	0.1 ± 0.2	− 0.1, 0.3
CG	0.2 ± 0.4	− 0.03, 0.5	− 0.2 ± 0.3	− 0.4, 0.1	0.03 ± 0.3	− 0.2, 0.3	0.1 ± 0.2	− 0.1, 0.3	0.01 ± 0.3	− 0.2, 0.2
KF (°)										
IG	−28.4 ± 6.7	− 33.2, − 23.6	− 29 ± 7.8	− 33.7, − 24.4	− 31.6 ± 10.5	− 37.3, − 26	−29.8 ± 10.2	−35.5, − 24.1	−29.5 ± 11.2	−36, − 23.1
EG	− 29 ± 7.4	− 33.8, − 24.1	−31.9 ± 7.2	− 36.5, − 27.3	−30.5 ± 5.1	− 36.1, − 24.8	−30.6 ± 7.5	− 36.3, − 24.9	−30.4 ± 9.4	− 36.8, − 23.9
CG	− 30.3 ± 8.2	− 35.1, − 25.4	− 31.8 ± 6.3	− 36.5, − 27.2	−32 ± 9.5	− 37.7, − 26.4	−32 ± 8.3	− 37.6, − 26.3	−32 ± 9.2	− 38.6, − 25.5
KA (°)										
IG	1 ± 2.7	−1.2, 3.1	0.7 ± 2.7	−1.6, 3.1	0.6 ± 1.8	−1.6, 2.7	1.7 ± 3.2	−0.3, 3.7	0.9 ± 1.4	−1.1, 3
EG	0.8 ± 3.9	−1.4, 3	0.4 ± 4.4	−2, 2.4	1.3 ± 4	0.8, 3.4	2.3 ± 2.8	0.3, 4.3	0.6 ± 2.8	−1.5, 2.6
CG	0.2 ± 3.4	−2, 2.4	−0.5 ± 3.5	− 2.6, 1.9	1.1 ± 3.6	−1, 3.2	1.2 ± 3.3	−0.8, 3.2	0.5 ± 4.4	−1.6, 2.5
KAM (Nm/kg)										
IG	0.03 ± 0.2	−0.1, 0.2	0.1 ± 0.2	−0.1, 0.2	0.03 ± 0.2	−0.1, 0.2	0.1 ± 0.2	−0.1, 0.2	0.03 ± 0.3	−0.1, 0.2
EG	−0.05 ± 0.2	−0.2, 0.1	− 0.1 ± 0.2	−0.2, 0.01	− 0.1 ± 0.2	−0.2, 0.04	− 0.1 ± 0.2	−0.2, 0.04	− 0.02 ± 0.1	−0.2, 0.1
CG	0.03 ± 0.2	−0.1, 0.2	−0.1 ± 0.1	− 0.2, 0.01	0.003 ± 0.1	− 0.1, 0.1	0.04 ± 0.2	− 0.1, 0.2	−0.02 ± 0.2	− 0.2, 0.1

Mean ± standard deviation (SD) and 95% confidence intervals (CI)

Hip flexion (HF), hip abduction (HA), hip abduction moment (HAM), knee flexion (KF), knee abduction (KA), knee abduction moment (KAM)

Implicit group (IG), explicit group (EG), Control group (CG)

**Table 3:** Descriptive statistics for all dependent measures by group across testing periods measured at peak

Box Drop-Jump Task	Pretest		Posttest 1		Posttest 2		Posttest 3		Retention	
	Mean ± SD	95% CI	Mean ± SD	95% CI	Mean ± SD	95% CI	Mean ± SD	95% CI	Mean ± SD	95% CI
HF (°)										
IG	32.6 ± 12.4	26, 39.2	35.4 ± 9.6	29.3, 41.4	36 ± 11.9	29.3, 42.6	38.5 ± 13.9	30.8, 46.1	31.8 ± 16.3	24.2, 39.5
EG	31.8 ± 9.6	25.2, 38.4	34.1 ± 10.1	28.1, 40.2	35.9 ± 9	29.2, 42.5	37.3 ± 11.6	29.7, 44.9	34.9 ± 6.8	27.3, 42.5
CG	34.1 ± 7.9	27.5, 40.7	36.9 ± 8.1	30.9, 42.9	37.9 ± 9.6	31.3, 44.6	38 ± 9.3	30.4, 45.6	36.1 ± 10.1	28.5, 43.7
HA (°)										
IG	−3.5 ± 4.2	−6.8, −0.2	−3.3 ± 3.4	− 6, −0.7	−4 ± 4.7	−7.1, −1	−5.2 ± 5.7	−9.1, −1.4	− 3.7 ± 4.6	− 7.4, − 0.1
EG	−6.6 ± 5.6	− 9.9, − 3.2	− 7.1 ± 4.7	− 9.8, − 4.5	−8.4 ± 3.5	−11.5, − 5.4	− 7.7 ± 5	−11.5, − 3.9	− 9.5 ± 5	−13.2, − 5.8
CG	− 7.6 ± 5.5	− 10.9, − 4.	− 6.8 ± 4	− 9.5, − 4.2	−4.8 ± 5.7	− 7.9, − 1.78	−6.3 ± 6.7	− 10.1, −2.5	− 5.5 ± 7.2	− 9.2, − 1.8
HAM (Nm/kg)										
IG	−1.3 ± 0.7	− 1.7, − 0.9	−1.4 ± 1	− 1.8, − 1	− 1.2 ± 0.5	−1.5, − 0.9	−1.2 ± 0.4	− 1.5, − 0.9	−1.2 ± 0.5	− 1.4, − 0.9
EG	−1.1 ± 0.6	− 1.6, − 0.7	−1 ± 0.2	− 1.4, − 0.6	−1.1 ± 0.4	− 1.3, − 0.8	−1.1 ± 0.6	− 1.4, − 0.8	− 0.9 ± 0.3	−1.1, − 0.7
CG	− 1.2 ± 0.7	−1.7, − 0.8	−1.1 ± 0.4	− 1.5, − 0.7	−1.1 ± 0.4	− 1.4, − 0.8	−1 ± 0.3	− 1.3, − 0.7	−1 ± − 0.3	−1.3 ± 0.8
KF (°)										
IG	− 102.3 ± 13.7	− 110.1, − 94.4	− 104.2 ± 14	− 114.3, − 94	−104.8 ± 18.5	− 116.8, − 92.8	−104.8 ± 15.9	− 114.9, − 94.6	− 102.7 ± 16.1	− 113.1, − 92.2
EG	−99.6 ± 8.6	− 107.5, − 91.8	−108.2 ± 16.6	− 118.4, − 98.1	− 109.3 ± 18.5	− 121.3, − 97.3	− 110.1 ± 14.6	− 120.2, − 99.9	−109.5 ± 14.1	− 120, − 99.1
CG	− 98.6 ± 13.5	− 106.4, − 90.7	− 102.7 ± 16.2	− 112.9, − 92.6	− 100.7 ± 18.5	− 112.7, − 88.7	− 103.5 ± 16.4	− 113.6, − 93.3	− 102.80 ± 17.9	− 113.2, − 92.4
KA (°)										
IG	−5.1 ± 2.5	−8.2, − 2.1	−4.2 ± 3.4	− 7.3, − 1.1	−5.35 ± 2.0	− 7.7, − 3	− 4.1 ± 4.5	−6.5, − 1.8	− 5.0 ± 3.0	− 7.2, − 2.9
EG	− 5.4 ± 5.5	− 8.4, − 2.3	− 6.7 ± 5.5	−9.8, − 3.6	− 4.4 ± 4.5	− 6.7, − 2.1	−2.1 ± 3.6	− 4.5, 0.3	− 7.1 ± 3.6	−9.3, − 5
CG	−4.4 ± 5.5	− 7.5, − 1.4	−6.2 ± 5.1	−9.3, − 3.1	−3.8 ± 3.7	−6.1, − 1.5	−3.3 ± 2.6	−5.7, − 0.9	−5.2 ± 3.3	−7.3, − 3.1
KAM (Nm/kg)										
IG	− 0.4 ± 0.2	− 0.6, − 0.3	− 0.5 ± 0.3	− 0.6, − 0.4	−0.4 ± 0.2	− 0.5, − 0.26	−0.4 ± 0.2	− 0.5, − 0.3	−0.4 ± 0.2	− 0.5, − 0.3
EG	−0.4 ± 0.2	− 0.1, − 0.2	−0.4 ± 0.1	− 0.5, − 0.2	−0.4 ± 0.2	0.5, − 0.3	−0.4 ± 0.2	− 0.5, − 0.3	−0.3 ± 0.1	− 0.4, − 0.2
CG	−0.4 ± 0.3	− 0.5, − 0.3	−0.4 ± 0.1	− 0.5, − 0.3	−0.4 ± 0.2	− 0.5, − 0.3	−0.4 ± 0.1	− 0.5 -0.2	−0.3 ± 0.1	− 0.4, − 0.3
vGRF (N)										
IG	2162.9 ± 436	1907.4, 2418.4	2212.2 ± 441	1975.5, 2448.8	2075.2 ± 473	1822.6, 2327.9	2059.8 ± 398	1811.5, 2308.2	2093.5 ± 404	1865.3, 2321.6
EG	2239.7 ± 304	1984.2, 2495.2	2194.7 ± 308	1958.1, 2431.4	2140.3 ± 338	1887.6, 2392.9	2091.9 ± 361	1843.5, 2340.3	2030.8 ± 340	1802.7, 2258.9
CG	2362.1 ± 427	1984.2, 2495.2	2179 ± 331	1942.3, 2415.6	2064.8 ± 342	1812.2, 2317.4	2031.8 ± 388	1783.4, 2280.2	2023.8 ± 303	1795.7, 2251.9

Mean ± standard deviation (SD) and 95% confidence intervals (CI)

Hip flexion (HF), hip abduction (HA), hip abduction moment (HAM), knee flexion (KF), knee abduction (KA), knee abduction moment (KAM)

Implicit group (IG), explicit group (EG), Control group (CG)

A main effect for time was also found for peak for hip flexion, hip abduction moment, knee flexion and abduction angle, knee abduction moment, and vGRF where *p* < 0.05 (Table 4). Hip flexion significantly increased from pretest to posttest-3 and decreased from posttest-3 to retention (*p* = 0.02). Furthermore, hip abduction moment was smaller at retention than posttest-1 (*p* = 0.05). In addition, knee flexion increased from pretest to posttest-2, posttest-3, and retention (*p* = 0.01). Knee abduction angle decreased from pretest and posttest-3 and then increased from pretest-3 to retention (*p* = 0.002). Knee abduction moment was significantly smaller from posttest-2 to retention (*p* = 0.01). Lastly, vGRF decreased from pretest to posttest-3 and to retention, and then from posttest-1 to posttest-3 and to retention (*p* = 0.01).

**Table 4:** Descriptive statistics for time main effect for all dependent measures at initial contact and peak

	Pretest		PST1		PST2		PST3		Retention	
	Mean (SD)	95% CI	Mean (SD)	95% CI	Mean (SD)	95% CI	Mean (SD)	95% CI	Mean (SD)	95% CI
Heel strike										
HF (°)	32.8 ± 9.8^a^	29.0, 36.6	35.3 ± 9.3	31.7, 38.9	36.9 ± 9.7^a^	33.2, 40.6	37.9 ± 11.3^a^	33.5, 42.3	34.1 ± 11.5^a^	29.7, 38.5
HA (°)	−8.2 ± 4.6	−9.9, −6.5	−7.5 ± 3.8	− 8.9, − 6.1	− 7.9 ± 4.9	−9.7, − 6.1	−8.3 ± 5.3	− 10.3, − 6.3	−8.2 ± 5.1	−10.1, − 6.3
HAM (Nm/kg)	0.09 ± 0.4^a^	−0.1, 0.2	−0.08 ± 0.4^a^	−0.2, 0.05	−0.02 ± 0.4^a^	−0.2, 0.2	0.05 ± 0.3	−0.1, 0.2	0.04 ± 0.3	−0.1, 0.2
KF (°)	−29.2 ± 7.2	−32, − 26.4	−31 ± 7	−33.6, − 28.2	− 31.4 ± 8.4	− 34.6, − 28.1	− 30.8 ± 8.5	− 34, − 27.5	−30.6 ± 9.7	− 34.4, − 26.9
KA (°)	0.7 ± 3.3^a^	−0.6, 1.9	0.2 ± 3.5^a^	− 1.2, 1.6	1.0 ± 3.2	−0.2, 2.2	1.7 ± 3^a^	0.6, 2.9	0.7 ± 3	−0.5, 1.8
KAM (Nm/kg)	0.003 ± 0.2	−0.07, 0.08	−0.05 ± 0.2^a^	− 0.1, 0.02	−0.02 ± 0.2	− 0.09, − 0.06	0.01 ± 0.2^a^	−0.06, 0.08	0.004 ± 0.2	−0.08, 0.07
Peak										
HF (°)	32.8 ± 9.8^a^	29.0, 36.6	35.5 ± 9.1	32.0, 38.9	36.6 ± 9.9	32.7, 40.4	37.9 ± 11.3^a^	33.5, 42.3	34.3 ± 11.5^a^	29.9, 38.7
HA (°)	−5.9 ± 5.3	−7.8, −4	−5.7 ± 4.3	− 7.3, − 4.2	− 5.8 ± 4.9	−7.5, − 4	−6.4 ± 5.7	−8.6, − 4.2	−6.2 ± 6.1	−8.4, − 4.1
HAM (Nm/kg)	−1.2 ± 0.7	− 1.5, − 1	− 1.2 ± 0.6^a^	− 1.4, − 1	− 1.1 ± 0.4	−1.3, − 1	−1.1 ± 0.5	− 1.3, − 0.9	−1 ± 0.4^a^	− 1.2, − 0.9
KF (°)	− 100.1 ± 13.9^a^	−104.7, − 95.6	− 105 ± 15.3	− 110.9, − 99.2	− 105 ± 18.2^a^	− 111.8, − 98	−106.1 ± 15.4	− 112, − 100.2	105 ± 15.9^a^	− 111, − 99
KA (°)	− 5.0 ± 4.6^a^	− 6.7, − 3.2	− 5.7 ± 4.7	− 7.5, − 3.9	− 4.5 ± 3.5	−5.8, − 3.2	−3.2 ± 3.7	− 4.6, − 1.8	− 5.8 ± 3.3^a^	− 7.0, − 4.5
KAM (Nm/kg)	− 0.4 ± 0.2	− 0.5, − 0.3	−0.4 ± 0.2	− 0.5, − 0.3	−0.4 ± 0.2^a^	− 0.4, − 0.3	−0.4 ± 0.2	− 0.4, − 0.3	−0.3 ± 0.2^a^	− 0.4, − 0.3
GRF (N)	2254.9 ± 389^a^	2107, 2402	2195.3 ± 352^a^	2059, 2332	2093.4 ± 377	1948, 2239	2061.2 ± 370^a^	1918, 2205	2049.3 ± 341^a^	1919, 2181

Mean ± standard deviation (SD) and 95% confidence intervals (CI)

Hip flexion (HF), hip abduction (HA), hip abduction moment (HAM), knee flexion (KF), knee abduction (KA), knee abduction moment (KAM)

Implicit group (IG), explicit group (EG), Control group (CG)

^a^Statistically significant results

**Fig. 2:**
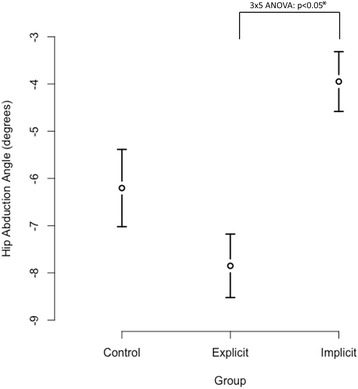
Visual depiction of group main effect for peak hip abduction angle of mean ± 2 standard deviations. Statistically significant difference between explicit and implicit groups, where implicit group is closer to neutral alignment

A statistically significant difference for group main effect (Table 5) was attained (*p* < 0.05). Group differences for hip abduction angle and knee abduction moment at initial contact were found. Specifically, for hip abduction angle the IF group had significantly lower (− 6.7 ± 4.0**°**) angle than the EF group (− 9.4 ± 4.1**°**), and for knee abduction moment the IF group was significantly greater (0.05 ± 0.2 Nm/kg) than the EF (− 0.07 ± 0.2 Nm/kg) group. At peak, there was a significant group effect for hip abduction angle and hip abduction moment (*p* < 0.05). Hip abduction angle for the IF group was significantly lower (− 3.5 ± 4.5**°**) than the EF (− 7.9 ± 4.7**°**) group (Fig. 2); for hip abduction moment, the IF group (1.1 ± 0.6 Nm/kg) was significantly greater than the EF (0.9 ± 0.4 Nm/kg) group. The control group main effect did not reach significance.

**Table 5:** Descriptive statistics for group main effect for all dependent measures at initial contact and peak

	EXP		IMP		CON		*p* (group)
	Mean (SD)	95% CI	Mean (SD)	95% CI	Mean (SD)	95% CI	
Heel strike							
HF (°)	34.8 ± 9.3	28.9, 40.6	35 ± 12.7	29.1, 40.8	36.5 ± 8.7	30.7, 42.3	n.s.
HA (°)	−9.4 ± 4.1^a^	−12.1, −6.7	− 6.7 ± 4^a^	− 9.4, − 4	−7.9 ± 5.5	− 10.6, − 5.2	0.02
HAM (Nm/kg)	−0.03 ± 0.4	−0.2, 0.1	0.04 ± 0.4	−0.1, 0.2	0.04 ± 0.3	−0.1, 0.2	n.s.
KF (°)	−30.4 ± 7.2	−35.4, −25.5	− 29.7 ± 9.1	−34.6, − 24.7	− 31.6 ± 8	− 36.6, − 26.7	n.s.
KA (°)	1.1 ± 3.6	− 0.8, 2.9	1 ± 2.4	− 0.9, 2.9	0.5 ± 3.5	− 1.4, 2.4	n.s.
KAM (Nm/kg)	−0.07 ± 0.2^a^	−0.2, 0.03	0.05 ± 0.2^a^	−0.06, 0.2	− 0.01 ± 0.2	−0.1-0.1	0.01
Peak							
HF (°)	34.8 ± 9.3	29, 40.6	34.8 ± 12.7	29, 40.7	36.6 ± 8.8	30.8, 42.4	n.s.
HA (°)	−7.9 ± 4.7^a^	−10.7, 5	−3.9 ± 4.5^a^	−6.8, − 1.1	−6.2 ± 5.8	−9.1, − 3.3	< 0.001
HAM (Nm/kg)	− 1 ± 0.4^a^	− 1.3, −0.8	−1.3 ± 0.6^a^	− 1.5, − 1	− 1.1 ± 0.4	−1.4, − 0.8	0.02
KF (°)	−107.3 ± 14.8	− 116.7, − 98	− 103.7 ± 15.1	− 113.1, − 94.3	− 101.6 ± 16	− 111, − 92.3	n.s.
KA (°)	−5.1 ± 4.8	− 7, − 3.3	−4.8 ± 3.1	− 6.6, − 2.9	−4.6 ± 4.1	− 6.4, − 2.7	n.s.
KAM (Nm/kg)	− 0.3 ± 0.2^a^	− 0.4, − 0.3	−0.4 ± 0.2^a^	− 0.5, − 0.3	−0.4 ± 0.2	− 0.5, − 0.3	n.s
GRF (N)	2139.5 ± 326	1937, 2342	2120.7 ± 418	1918, 2323	2132.3 ± 371	1930, 2335	n.s.

Mean ± standard deviation (SD) and 95% confidence intervals (CI)

Hip flexion (HF), hip abduction (HA), hip abduction moment (HAM), knee flexion (KF), knee abduction (KA), knee abduction moment (KAM)

Implicit group (IG), explicit group (EG), Control group (CG)

^a^Statistically significant results; n.s.-not significant results

## Discussion

The purpose of this study was to examine the short and long-term effects of reduced feedback frequency on motor learning. We evaluated lower extremity biomechanics during landing at the hip and knee joints during initial contact and peak, as well as vGRF. Our results partially supported our hypotheses; we found a statistically significant main effect between groups (implicit and explicit) for hip abduction and knee abduction moment at initial contact, and hip abduction and hip abduction moment at peak; supporting our hypothesis that implicit would have greater improvements than the explicit group. However, contrary to our hypothesis, the implicit and explicit group did not significantly improve when compared to the control group. It is possible that reducing implicit feedback may in fact hinder the benefits that implicit feedback provides.

Previous studies supported the use of both implicit and explicit feedback in ACL injury prevention training. Our results showed no significant interaction between group and time for any dependent measure. This contrast with research reporting that video feedback in conjunction with verbal instruction led to safer landing during a single-leg drop jump task in ACLR females (Tsai and Powers 2013). They found that participants improved their landing technique by increasing hip and knee flexion angles, decreasing peak tibiofemoral compressive force and vGRF after just one training session. In contrast, our participants were healthy and physically active individuals who may already have been at lower risk for ACL injury. It is conceivable that our participants had limited room to improve their landing mechanics and present significant improvements when compared to the control group. Recent studies suggested that augmented feedback may produce short and long term changes in landing technique, lower vGRF, and possibly reduce the ACL injury risk during a drop-jump task (Myer et al. 2013; Etnoyer et al. 2013; Munro and Herrington 2014). Myer and colleagues reported that combination of video and explicit verbal feedback significantly reduced high risk injury landing mechanics after drop-jump in high school female athletes; (Myer et al. 2013) our explicit verbal instructions were similar to those in Myer’s study. However, the explicit group participants evaluated their performance without specific performance perspective from the investigator. It is possible that our participants did not have enough information to alter their lower extremity biomechanics like those in Myer et al. study. Drop-jump is a multi-joint movement that includes complex neuronal control mechanisms before jump, during jump, and during take-off phase (Malfait et al. 2016). It is possible that participants could not recognize and correct false movement because of complexity of a task. Future research should focus on splitting the drop-jump task to concentric and eccentric components and analyze each part separately. Contrary to our results, several studies reported long term changes in lower body mechanics when using augmented feedback (Laufer et al. 2007; Etnoyer et al. 2013; Benjaminse et al. 2015). For example, Etnoyer et al.(Etnoyer et al. 2013) found that during a drop-jump task, participants who received augmented feedback increased hip flexion and decreased hip abduction angle right after receiving the feedback and maintained changes 1 month after in the retention. Similar results reported Benjaminse et al. (Benjaminse et al. 2015) who observed that participants maintain changes in lower extremities mechanics during a cutting-task 1 and 4 weeks after feedback was withdrawn. Laufer et al. observed increases in overall and anteroposterior stability right after training and after 48 h without practice (Laufer et al. 2007).

Remarkably, we identified two patterns of variables change across time. In the first pattern, variables improved throughout the intervention, but returned close to or at baseline values at post-test 3 and retention. We observed this pattern in hip abduction/flexion and knee abduction angle at peak, and hip abduction/flexion angle, hip abduction moment, knee flexion angle, and knee abduction moment at initial contact. These findings are similar to results from Onate et al. study who reported that both feedback and control group participants decreased vGRF and increased peak knee flexion angle and knee angular displacement (Oñate et al. 2005). We noticed some minor adaptation during the training, between posttest 1 and 2, but once feedback was removed, the biomechanical measures returned to the baseline normal values. Our results are partially comparable with those from Etnoyer et al. who observed that hip flexion angles at initial contact and peak in self-feedback group increased when using video and verbal feedback during a drop-jump task but came to initial values in retention (Etnoyer et al. 2013). All participants in our study increased hip flexion angle from pretest to posttest but did not retain changes at the retention assessment. Hip flexion angle gradually increased from pretest to posttest 3, but in the retention period it returned to the pretest values. This result suggests that limited changes in lower body mechanics were not retained 1 month after the training. Similar pattern was observed for knee abduction angle at peak and initial contact. Participants decreased knee abduction angle from pretest to posttest 3, yet at retention they adopted similar biomechanical patterns as at pretest. Anderson et al. suggested that if explicit feedback is provided too frequently, participants might develop an over-reliance on the feedback (Anderson et al. 2005). It is possible that the frequency of the feedback in our study was not adequate to lead to long-term changes in learning landing technique. Our participants could overlook relevant sensory information that is native to the task. Several authors proposed lowering feedback frequency as a solution to this reliance (Schmidt 1991; Lai and Shea 1999). Reducing relative feedback frequency should decrease participants’ dependence on feedback and provide the participants with an opportunity to internalize the new movement patterns (Wulf et al. 2002). It is possible that our explicit feedback was excessive and led participants’ reliance on feedback even though we progressively lowered feedback frequency. These findings may indicate that alternative frequency reduction strategies may be necessary for the explicit group after acquisition phase, to decrease participants’ reliance on feedback. It is possible that participants continued to rely on feedback and most likely did not develop an error detecting system and consequently performed at pretest levels during the retention assessment.

Some of our variables improved during the 6-week intervention period and maintained changes during the retention assessment. All participants improved peak hip/knee abduction moment and peak knee flexion angle and decreased peak vGRF. Previous studies identified these biomechanical parameters as risk factors for ACL injury (Munro and Herrington 2014; Kim et al. 2015). Our participants gradually decreased hip and knee abduction moments and maintained the same pattern once feedback was removed. Previous research has implemented verbal and video feedback to modify knee/hip abduction moments and vGRF to possibly lower ACL injury rates (Myer et al. 2013; Munro and Herrington 2014). Etnoyer reported that participants retain learned landing technique 1-month after explicit feedback was provided (Etnoyer et al. 2013). We observed similar results in hip/knee abduction moment and knee flexion angle at peak, and vGRF where we noticed a positive change during the 10-week period. From a theoretical perspective, implicit and explicit feedback presented positive outcomes in altering lower extremity biomechanics that are linked with the risk of ACL injury (Gokeler et al. 2013; Benjaminse et al. 2015). In fact, many ACL injury prevention programs contain both feedback modalities used in prevention and rehabilitation that can accelerate motor learning and enhance performance (Stroube et al. 2013; Munro and Herrington 2014). A plausible explanation for our results is that the type of feedback, in isolation, was not sufficient to produce significant motor pattern changes in a healthy population. Although all three groups (even control group) improved over time, the implicit group showed an overall positive change, possibly reducing the risk for ACL injury and explicit feedback showed the opposite trend. Those findings are in line with earlier research where participants degrade technique when using explicit feedback (Wulf and Su 2007; Benjaminse and Otten 2011).

Recent research suggests that feedback that induces an external focus (implicit feedback) could possibly facilitate motor learning better when provided on a higher frequency (Wulf et al. 2010; Welling et al. 2016). Welling et al. (Welling et al. 2016) reported that participants improved LESS score in drop-jump at posttest (same day) and retention test (1 week after) when giving an implicit feedback after every jump. Our results showed a trend of improving variables in all groups. However, we found a greater improvement in hip abduction angle (both initial contact and peak), hip abduction moment, and knee abduction moment at initial contact in implicit feedback group. Implicit group participants in our study did not reach significance from pretest to posttest as previous reported (Welling et al. 2016). Other modes of neuromuscular training (such as core strengthening, balance, flexibility) have clear benefits for motor learning of safer landing and should be included in the injury prevention programs.

## Conclusions

Prior injury prevention programs have presented video and augmented feedback as an important factor in prevention and rehabilitation that can accelerate motor learning and enhance performance. Our results showed that implicit feedback induce positive change in landing mechanics while explicit feedback showed the opposite trend. Future research should focus on exploring the optimal frequency of implicit feedback on learning safer landing. While the guidance hypothesis suggests that feedback should be lowered to prevent dependence of feedback, it is possible that participants will not benefit from reduced implicit feedback if not enough feedback is presented in early phases of motor learning, in the acquisition and transfer phases. Implicit feedback should be frequent in the beginning, so participants have enough time to internalize the new movement pattern. This becomes particularly important for complex movements such as learning safe landing after a drop-jump. If implicit feedback is reduced too quickly, participants may not reach automatic processing of the correct movement in the brain, which further leads to poor performance in the retention. Our feedback frequency in implicit group may have not been sufficient to induce motor changes. It is also likely that our participants had less room for improvement, since they demonstrated lower risk (Hewett 2005) jump-landing mechanics during the pretest session. We suggest that further research should explore the influence of implicit feedback in high-risk population. The other modes of neuromuscular training would possibly add to benefits of implicit feedback and should be included in programs for preventing the future injuries with healthy population. Future research should explore which of these modalities or whether any specific combination leads to improved performance and good transfer of learned patterns in retention period. The understanding of this phenomenon would certainly help health professionals to choose the best strategy when planning injury prevention programs.
